# Increase the elongation at break of poly (lactic acid) composites for use in food packaging films

**DOI:** 10.1038/srep46767

**Published:** 2017-05-03

**Authors:** Ahmed M. El-hadi

**Affiliations:** 1 Department of Physics, Faculty of Applied Science, Umm Al-Qura University, Al-Abidiyya, P.O. Box, 13174, Makkah, 21955, Saudi Arabia; 2 Higher Institute of Engineering and Technology, Department of Basic Science, El Arish, North Sinai 9004, Egypt

## Abstract

Poly (3-hydroxy butyrate) (PHB), cellulose nano crystal (CNC) and a plasticizer (TBC) are mixed together with PLLA with the aim to increase the elongation at break for use in the food packing sector. Spherical (CNC) and fibril nano crystal (CNF) were prepared by hydrolysis of microcrystalline cellulose (MCC) in distilled water, and then stirred using a magnetic stirrer for 15 days and ultrasonic treatment without using any acids as green method. The morphology, thermal, and mechanical properties were studied using POM, DSC, WAXD, SEM and tensile testing, respectively. DSC demonstrated that the addition of PHB, CNC and TBC to PLLA matrix lead to reduce T_g_, T_CC_ and T_m_ than pure PLLA. FT-IR verified that the carbonyl group C=O appeared broad and some peaks in the PLLA composites 5, 6 and 7 shifted from 3.98 × 10^8^ to 4.07 × 10^8^ Hz, at 3.54 × 10^8^ to 3.44 × 10^8^ Hz, at 3.19 × 10^8^ to 3.13 × 10^8^ Hz. Mechanical testing shows that pure PLLA is brittle, and the elongation at break of PLLA composites reaches up to 205%, making it suitable to use in food packaging.

Recently, interest in renewable resources and the production of bio-polymers has increased worldwide in response to many environmental and economic issues, such as rising oil prices and carbon dioxide emissions that result from burning plastic waste. Therefore, the disposal of packaging from petrochemical-based plastic is a major concern for governments and scientists in many countries. The amount of bio-plastic produced in the world is greatly less in comparison to petrochemical plastic^1^. Sugar and cellulose, in their various chemical forms, are potential alternatives to crude oil and offer a possible avenue for creating work opportunities in several countries. These materials can be used to produce biopolymers like PHB, PLLA and cellulose in polymer blends. For instance, lactic acid is derived from renewable resources, such as molasses, by fermentation^2^ and the lactic acid can then be polymerized to poly (lactic acid) by polycondensation or ring opening polymerization^3^. The resulting PLLA is biodegradable aliphatic polyester, and it is nontoxic to people and the environment. PLLA is an optically active, crystallizable thermoplastic. PLLA can be used in the food packing sector, in surgical sutures, and in drug delivery systems^4^,^5^. Pure PLLA has poor processing like air blown extrusion, because PLLA has lower shear and elongation properties^6^. The novelty of this paper is to solve the problem of brittleness of PLLA in order to use in food packing film. The physical and thermal properties of PLLA depend on its crystal structure, crystallinity, and morphology^7^,^8^,^9^. The reasons of brittle of PLLA; first the glass transition temperature (T_g_) is 60 °C; thus, the polymer chains are rigid and inflexible near room temperature; Second, PLLA has low nucleation density, therefore it has slow crystallization rates leading to the formation of large spherulites. These reasons have a negative effect on the mechanical properties. In order to overcome these problems and improve the elongation at break of PLLA, PHB, CNC and TBC with certain ratio were mixed together by solvent casting and then melting by hydraulic hot press to manufacture biocomposites film. Many studies have focused on improving the crystallization and mechanical properties of PLLA by attempting to copolymerize the lactide with another monomer^10^,^11^ or physically blending PLLA with immiscible biopolymers such as polycaprolactone (PCL)^12^, poly (butylene succinate) (PBS)^13^, poly (propylene carbonate) PPC^14^, and PHB^15^,^16^,^17^,^18^,^19^,^20^,^21^,^22^. PHB may be added to PLLA to accelerate the crystallization of PLLA; PHB is a fast crystallizing biomaterial. PHB and PLLA have similar chemical structures and their melting points 175 °C and 170 °C, respectively. It is noted, that PHB (10–20%) is miscible with PLLA^15^,^16^.

Micro cellulose crystals (MCC) and Cellulose nanocrystals (CNC) are a type of biodegradable nano-filler that has been added to PLLA matrix as bio nucleation and reinforcement. In recent years, the application of CNC as a bio filler in polymer composites has attracted expansive interest because of its’ renewable nature. Cellulose nano crystal (CNC) may be used as bio-nuclei for PLLA; it should increase the number of spherulites crystallizing and result in a reduction of crystal size. MCC is typically found as a powder with particles between 20 and 80 μm in size. As indicated previously. Natural fibers, such as rice straw, rice husk, oil palm, hemp, flax, jute, kenaf and other agricultural residues, have many advantages. These advantages include low cost, high performance, low weight, renewable and biodegradability relative to petrochemical fibers, such as glass fiber or carbon fiber. Numerous studies have concentrated on developing biodegradable composites by mixing PLLA with a natural fiber. This mixing includes flax, kenaf, jute, abaca, sisal fiber, wood fiber, bamboo fiber, newspaper fibers, microcrystalline cellulose MCC^23^,^24^,^25^,^26^, cellulose whiskers^27^,^28^, lignin^29^, carbon nanospheres^30^ and rice straw^31^, cellulose nano crystal (CNC)^32^,^33^,^34^,^35^. It is well known that plasticizers lead to increase the flexibility, to the reduction of the glass transition temperature, to increase the elongation at break and reduce the tensile strength of polymers. Another way to improve the elongation at break of PLLA is the use of plasticizers^36^,^37^,^38^,^39^,^40^,^41^,^42^,^43^,^44^,^45^,^46^,^47^,^48^,^49^,^50^ to reduce the glass transition temperature. These plasticizers must be non-toxic and biodegradable. There is one important condition to use PLLA film in the food packaging process by deep drawing article. This film must be high elasticity at room temperature, high transparency, and low crystallization. Small amount of PHB are used to improve the crystallization behavior and reduce the crystal size of PLLA^17^,^51^ as a bio-nucleation. The addition of plasticizer decreases glass transition temperature (T_g_) from 60 °C to 10 °C in blend^51^. There are a large number of papers in this field that add only PHB and PLLA blend or CNC and PLLA blend, or MCC and PLLA blend or PLLA and plasticizers blend, as previously mentioned in the literature. They studied the miscibility, morphology and crystallization behavior et cetera^6^,^15^,^45^. It is desirable to further develop a biodegradable polymer which is economically and environmentally friendly; in this work, biodegradable additives were evaluated to produce a biopolymer-based composite with improved flexibility, while maintaining its green properties. The objective of this study was to develop useful biodegradable thermoplastic that can be used for food packing application by introducing PHB, CNC or MCC with plasticizers together as biodegradable additives in PLLA matrix. The influence of plasticizer, PHB, CNC on the thermal properties, morphology, crystal structure, spherulite size, and mechanical properties of the PLLA was investigated. In this paper the physical interaction between PHB, CNC, TBC and PLLA has been studied, as well as the preparation of CNC as new green method.

## Results and Discussion

### DSC Analysis

**Figure 1:**
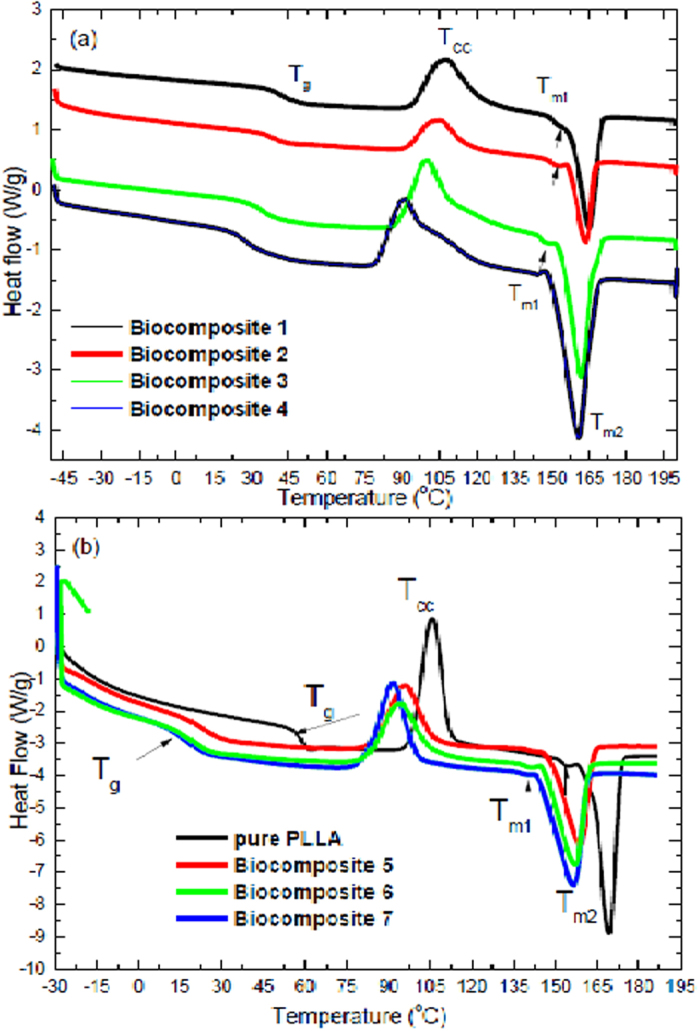
DSC second heating scan (**a**) biocomposites 1, 2, 3 and 4; (**b**) pure PLLA and biocomposites 5, 6, 7.

The second heating curves of the biocomposites 1, 2, 3 and 4 are shown in Fig. 1a. The addition of PHB, MCC and TCB to PLLA, resulted in a lower T_g_, T_CC_ and T_m_ than those associated with pure PLLA (Fig. 1b). From the DSC results, this can be calculated using the glass transition temperature (T_g_), cold crystallization temperature (T_cc_) and melting points (T_m1_ and T_m2)_. It can be observed that the T_g_, T_cc_ and T_m_ peaks shift towards lower temperatures with the addition of PHB, MCC, and TBC. α 1 shows that the T_g_ decreased from 60 °C for neat PLLA to 43 °C for biocomposite 1, to 38 °C for biocomposite 2, to 36 °C for biocomposite 3 and to 30 °C for biocomposite 4. Similarly, the T_m_ decreased from 168 °C for pure PLLA to 160–165 °C for all biocomposites. The T_cc_ increased from 107 °C for pure PLLA to 106 °C for biocomposite 1, 103 °C for biocomposite 2, and 100 °C for biocomposite 3, but decreased to 83 °C for biocomposite 4.

The second heating curves of pure PLLA and the biocomposites 5, 6 and 7 are shown in Fig. 1b. for pure PLLA showed one melting peaks at approximately 170 °C, but its biocomposites 5, 6 and 7 two peaks. The melting temperatures of composites were lower than that pure PLLA. It was detected that the adding of PHB, CNC and plasticizer in PLLA effected the T_g_, T_c_ and T_m_ during the second heating. The addition of plasticizer resulted in a lower T_g_. The DSC results can be calculated, the T_g_, T_cc_ and T_m1_ and T_m2_. The T_g_ of PLLA was affected by the addition of various contents of plasticizer. Table 1 shows that the T_g_ decreased for biocomposite 5, 22 °C for biocomposite 6, 16 °C for biocomposite 7 and 14 °C. The T_cc_ was 107 °C for pure PLLA, 96 °C for biocomposite 5, 94 °C for biocomposite 6, 90 °C for biocomposite 7. Similarly, the T_m_ decreased from 168 °C of pure PLLA to 160–165 °C for all biocomposites. In both Fig. 1a and b and Table 1 show that the T_m_ was reduced to 164 °C for 159 °C for biocomposite 5, 157 °C for biocomposite 6 and 156 °C biocomposite 7. This corresponds to two lamella thicknesses; the smaller one melts at a low temperature and the larger melts at a higher temperature. The reduction of T_m_ is a typical phenomenon for miscible blends containing a crystalline polymer^17^,^20^,^51^. The cold crystallization peak of PLLA is shifted to lower temperatures with the addition of PHB beside CNC. Both PHB and CNC are also effective bio-nucleation agents for PLLA, therefore a large reduction of T_cc_ in composites 5, 6 and 7 in comparison with pure PLLA. The composites 5, 6 and 7 are crystallized at temperatures between 80–90 °C. By the addition of PHB, CNC and TBC, the cold crystallization temperature (T_CC_) is moved from 106 °C to 84 of all PLLA composites. It can be concluded that the DSC results, the addition of PHB, MCC and TBC in PLLA matrix is used to reduce the glass translation temperature (T_g_) from 60–28 °C and to move the crystallization temperature (T_cc_) from 106–90 °C. But the addition of PHB, CNC and TBC to PLLA matrix to reduce the glass translation temperature (T_g_) from 60–14 °C and to move the crystallization temperature (T_cc_) from 106–84 °C to more in comparison to pure PLLA. Therefore the physical properties of PLLA has improved.

### Polarizing optical microscopy analysis

**Figure 2:**
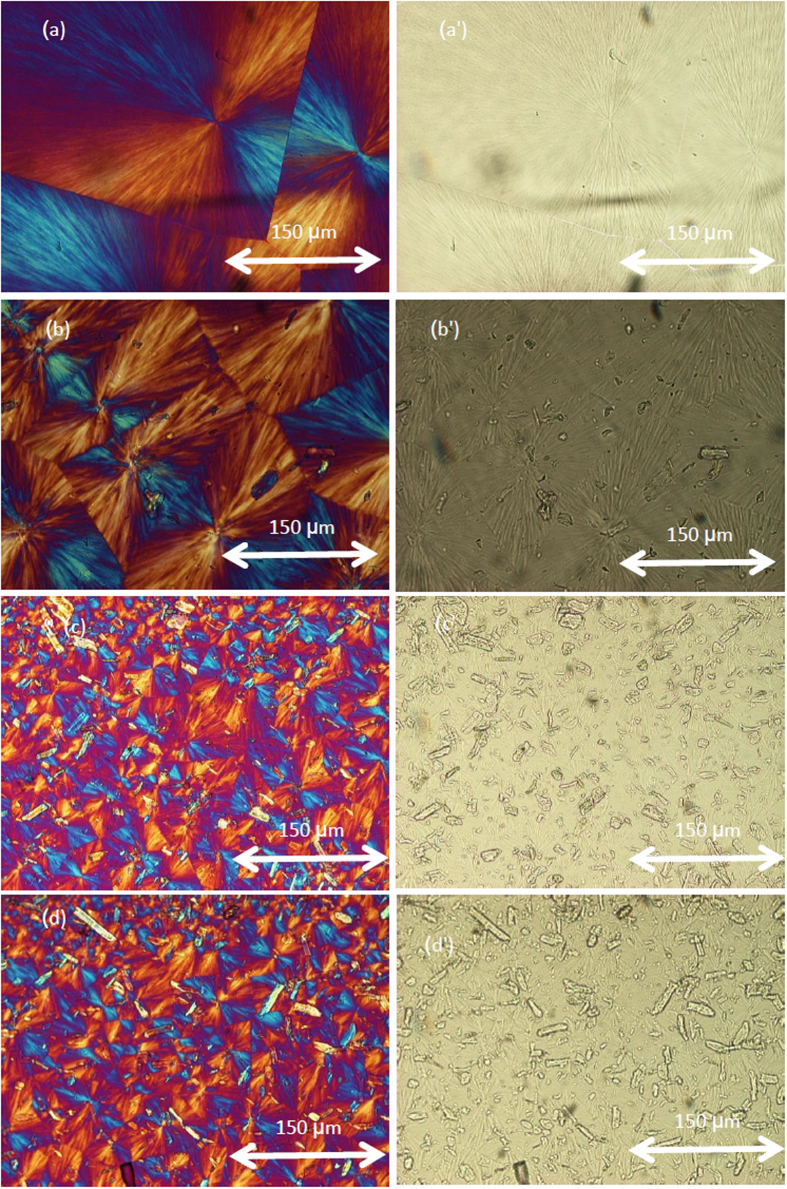
Polarized optical microscopy of spherulite of pure PLLA and its composite 1, 2 and 3 crystallized isothermal at 110 °C with optical polarizer (**a**) Neat PLLA, (**b**) biocomposite 1, (**c**) biocomposites 2, (**d**) biocomposite 3; without optical polarizer (a′) Neat PLLA, (b′) biocomposite 1 (c′) biocomposite 2, (d′) biocomposite 3.

**Figure 3:**
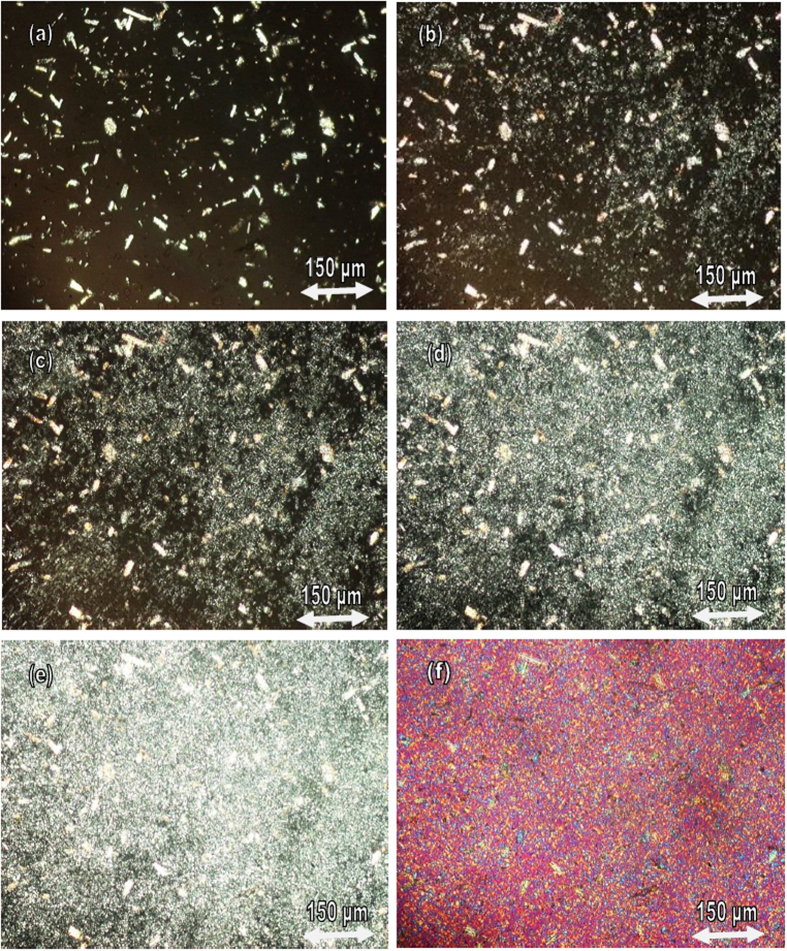
Polarized optical microscopy of spherulite of composite 7 crystallized isothermal at 80 °C after different time (**a**) 0, (**b**) 2 min, (**c**) 3 min, (**d**) 5 min, (**e**) 7 min, (**f**) 10 min. using a tinted plate (λ/4).

It is well known that materials with large spherulites are more brittle than those with fine spherulites with the same percentage of crystallinity. It was found that the size of spherulite is bigger and more cracks are formed after annealing at 160 °C (180 min.)^52^. Figure 2 show the optical micrographs of spherulites of samples of pure PLLA and composites 1. 2 and 3 that were obtained during isothermal crystallization at 110 °C. Figure 3(a) shows the molten polymer as a dark amorphous film. Numerous white MCC spots were observed in the PLLA matrix after melting, indicting good dispersion of the additive in the PLLA matrix.

High contents MCC in the PLLA matrix led to smaller spherulite structures; the growth of the PLLA spherulites was hindered and this suggests that MCC deters the expansion of spherulites. The crystal morphology of these composites revealed a typical spherulitic structure with a diameter of approximately 100–50 μm and the number of spherulites were produced and increased; the spherulites were much smaller than those produced with pure PLLA (Fig. 2a). It is known that pure PLLA exhibited a lesser number of large spherulites. It is important to understand the suitable melting and crystallization conditions for poly lactic acid (PLLA). Similar to other semi-crystalline polyesters, PLLA can be manufacture into either the amorphous or crystalline morphology by changing melting temperature processing and crystallization conditions (fast cooling we have amorphous PLLA, its glass transition temperature near 60 °C, it can never crystallization below this temperature, any crystallization process near the glass transition temperature cannot occur, but when we increase the annealing temperature to 90–160 °C, it turns into crystalline materials. Below 60 °C PLLA is amorphous, rigid and brittle. PLLA is a semi-crystalline polymer and by choice the bio nucleating agent like PHB and choice of the temperatures of melting and crystallization, one can obtain amorphous or crystalline morphology. It is acceptable to obtain PLLA in the amorphous morphology. Amorphous morphology is achieved by not adding any nucleating agents and fast cooling. By adding PHB as bio nucleation agent, it has crystalline part.

If the melting processing temperatures is higher than 180 °C, PLLA stick in the frame and becomes very soft and flexible and take longer time to leave the frame, at the end, PLLA become brittle. My arrangements in this paper, are how one can improve the crystallization behavior of PLLA without using annealing process.

It is concluded that PHB and CNC act as the heterogeneous nucleation agents for PLLA, the T_CC_ shifts from 107 °C to 84 °C for the composite 7, this means that the crystallization of PLLA became easier due to very fine dispersion of PHB and CNC into PLLA matrix. Figure 3(a–f) show micrographs of composite 7. The addition of PHB and CNC resulted in a decrease in the final size of spherulites in the PLLA biocomposites. One can see that the CNC, shown as bright spots, are homogeneously dispersed in the PLLA matrix without any visible agglomerates. It is concluded that the addition of CNC and PHB to PLLA lead to increase the number of spherulites with diameter less than 1 μm and the crystallization of PLLA is accelerated, therefore the elongation has improved than adding MCC. There is a clear difference between the MCC and CNC in terms the effect of the crystals size of PLLA, and mechanical properties. CNC is certainly better more than MCC.

### Study the morphology of CNC by TEM

**Figure 4:**
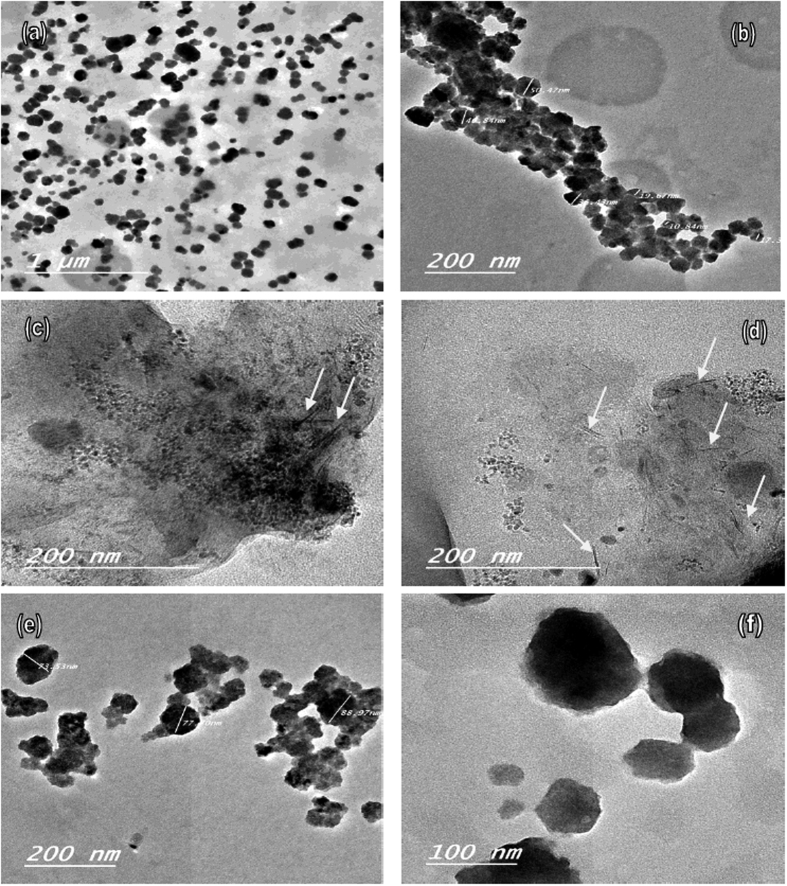
TEM images of spherical cellulose nanocrystal (CNC) in PLLA Matrix with different sizes.

The TEM images of CNC samples after preparation are shown in Fig. 4. It is known that the MCC particles have a rod-like fiber morphology. The average particle length of MCC was about 20–80 μm; after hydrolysis, the particle size of MCC decreased to 10–90 nm and the shape of the particles changed from micro rod to nano spherical and fibril. It was observed that CNC particles and CNF were spherical and fibril particles with nano dimensions; the average diameter of spherical CNC and CNF observed in a PLLA matrix by TEM was about 10–100 nm. CNC aggregates through the strong hydrogen bonds between the single cellulose crystallites, although single nanoparticles are sometimes detected, i.e. many small single particles and fibril collected together and form like fibril and spherical shape (Fig. 4c and d). Similar results were found in refs 53, 54, 55, 56, 57 for spherical cellulose nanocrystals with chemical hydrolysis of cellulose fibers. TEM investigation of biocomposite 7 showed well-dispersed spherical nano particles in a PLLA matrix. It is found both fibril like and spherical cellulose nano crystals by water hydrolysis using a magnetic stirrer and ultrasonic treatment.

### Crystalline structure analysis

**Figure 5:**
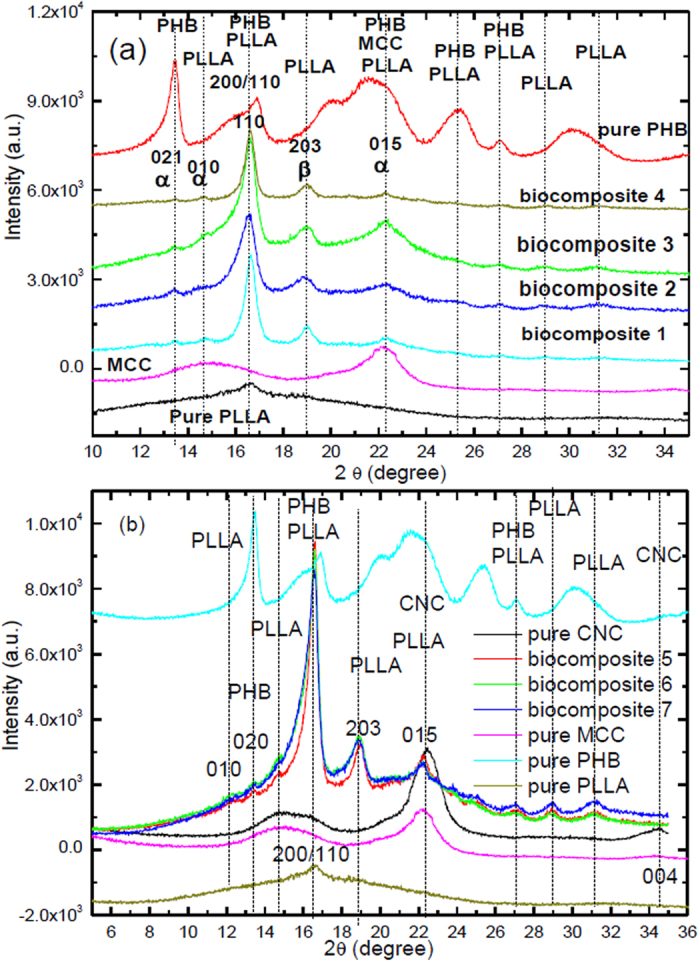
(**a** and **b**) WAXD spectra of PLLA and its biocomposites.

Figure 5a and b shows WAXD patterns of PLLA and its composites. Pure PLLA crystals are known to be typical orthorhombic^15^,^34^,^58^,^59^,^60^. A broad amorphous peak for PLLA at 2θ = of 16.6° is observed with additional small crystalline diffraction peaks. After addition of PHB, MCC or CNC and TBC to the PLLA matrix, two strong crystalline peaks appeared at 2θ of 16.6° and 22.4° corresponding to the (110)/(200) and (015) reflections, respectively. Figure 5b shows CNC with peaks at 2θ of 16.6, 22.5, and 34.5° assigned to planes (110), (200), and (004), which are believed to represent the typical cellulose structure. The 22.6° peak of the (200) plane of CNC is more observable than that of MCC. This indicates a higher perfection of the crystal lattice in the (200). The peak at 2θ of 14.7° for the (110) plane also became more intense for CNC in comparison to MCC and separated from the (110) reflection at 2θ of 16.4°. It is detected that the CNC peaks are broader than the MCC peaks, which confirms that the nanocrystals are more crystalline than MCC. Two overlapped peaks for CNC at 2θ = 14.8° and 16.6°, that is assigned to (101) and (101-) planes. We observed that the intensity peak of CNC at 2θ = 34° is larger comparison with MCC peak. The peak at 16.6° showed sharpening due to the presence of PHB and different content of CNC; this means that PLLA composites 5, 6 and 7 have some structural rearrangement. All of these peaks are typical diffraction peaks of the α forms of PLLA^15^, while the peak observed at 2θ at 19° is a reflection of the (203) and typical of the β form. The intensities of both the α and β crystal forms of the composites were higher and sharper than those observed for pure PLLA. The new small peak appeared at 2θ at 13.5° which indicates that PHB has changed and improved on the crystallinity of PLLA. The intensity of the crystalline peaks of PLLA increased with the additions of PHB and CNC to the composites compared to pure PLLA at room temperature. The inter-planar spacing (d) and crystal size can be calculated using the Bragg and Scherrer formulas:


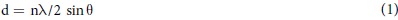



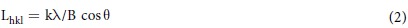


Where d is the spacing between the diffracting planes, *λ* is the wavelength of the x-ray (1.54 × 10^−10^ m), θ is the Bragg angle, L_hkl_ is the thickness of crystallite where h, k, and l are the Miller indices, K is the constant dependent on crystallite shape (0. 94), and β is the full width at half max (FWHM) or the integral breadth. Table 2 summarizes the structural parameters of the crystal, determined based on the WAXD patterns. The d spacing calculated for various peaks of the PLLA composites showed little change compared to those observed with pure PLLA. Although the thickness of the α form crystals at (110) increases from 23.7 to 30.5 nm in composites from 1 to 7. In addition, the thickness of β form crystals at (203) increases from 19.53 to 21.5 nm in the composites from 1 to 7.

### FT-IR spectroscopy analysis

**Figure 6:**
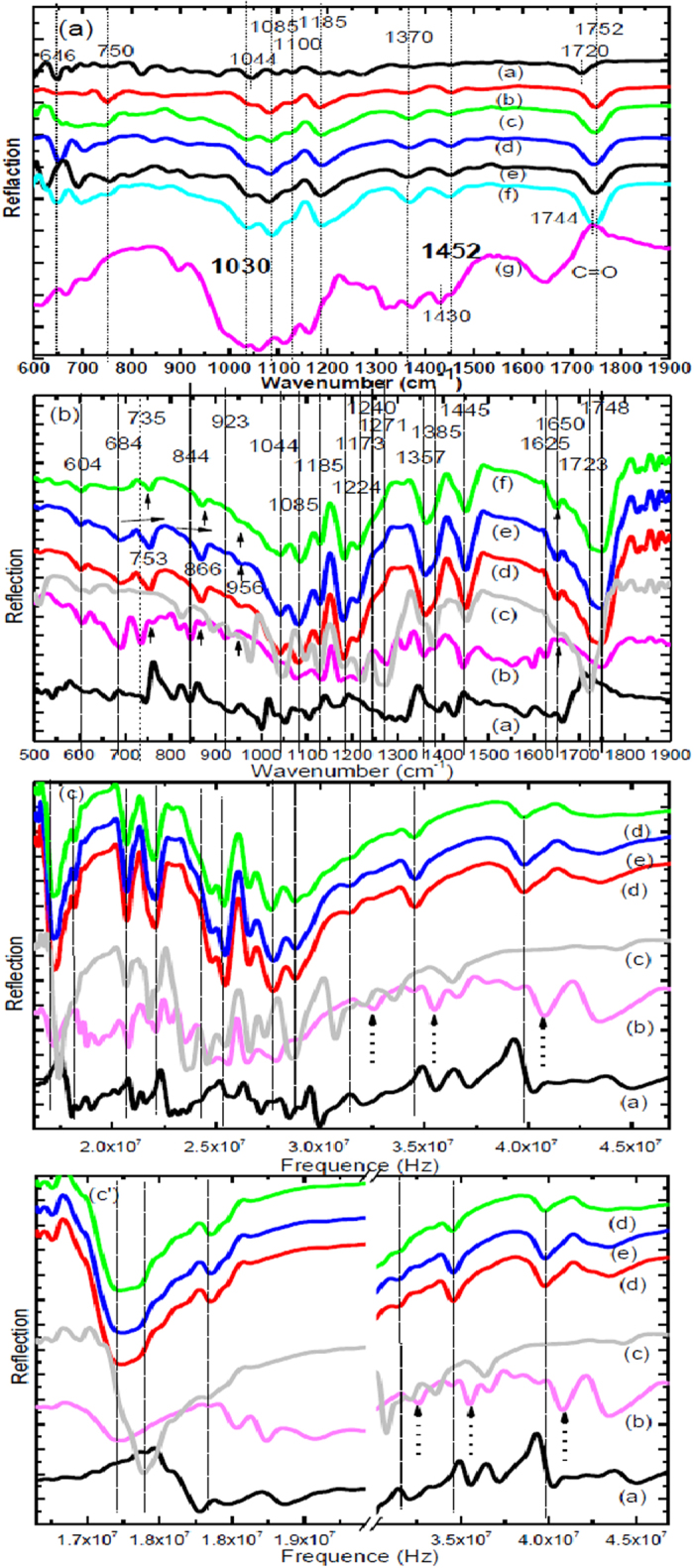
FT–IR spectra of (**a**) (a) PHB, (b) PLLA, (c) bioomposite 1, (d) bioomposite 2, (e) bioomposite 3, (f) bioomposite 4, (g) MCC; (**b**) (a) CNC, (b) pure PLLA, (c) pure PHB (d) biocomposite 5, (e) biocomposite 6, (f) bioomposite 7. [**c**, c′] FT–IR spectra of (a) CNC, (b) pure PLLA, (c) pure PHB (d) biocomposite 5, (e) biocomposite 6, (f) bioomposite 7.

Figure 6(a) shows the FTIR spectra of the pure PLLA, PHB, MCC and their composites in the region 800–1900 cm^−1^. The strong peak of the C=O ester band of PHB, PLLA and MCC appeared at 1723, 1752 and 1650 cm^−1^, respectively. By adding PHB, MCC and TBC, the peaks in PLLA moved to a lower wave number with composites 1, 2, 3 and 4, (1749, 1747, 1745 and 1744 cm ^−1^), respectively. In case of PLLA and PHB blends 15,60. It has been observed by the FT-IR spectrum, the band at 1723 cm^−1^, due to C=O stretching mode of the crystalline part of PHB. This peak appeared in the composites with very small shoulder. the carbonyl group centered at 1750 cm^−1^, due to the crystalline carbonyl stretching of PLLA and the peak at 1744 cm^−1^, assigned to C=O amorphous carbonyl vibration. The peak centered at 1740 cm^−1^ which is assigned to C=O stretching mode of the amorphous PHB^17^,^18^,^20^,^60^.

FTIR spectra of the pure PLLA, PHB, CNC and their composites in the region of 600–1900 cm^−1^ is shown in Fig. 5b. The band at 1723 cm^−1^ due to the C=O stretching mode of the crystalline part of PHB has been observed by FTIR^59^,^60^; this peak appeared in the composites with a very small shoulder. Additionally, there is a broad band centered near 1740 cm^−1^ which is assigned to C=O stretching mode of the amorphous PHB^20^. It is found that pure PLLA has a main peak at 1744 cm^−1^ due to C=O amorphous carbonyl vibration with a small shoulder attributed to the C=O crystalline carbonyl vibration. The band at 1224 cm^−1^ was assigned to the C–O–C stretching modes of the crystalline state and the band at 1185 cm^−1^ was attributed to the C–O–C of the ester group of the amorphous part of the PLLA. The two peaks at 1085 and 1044 cm^−1^ were attributed to C-O bond stretching of the C-O-C group, respectively, in PLLA and the composites. The band at 1452 cm^−1^ is due to CH_3_ of PLLA. The band at 1385 cm^−1^ is assigned to the symmetric deformation mode of the CH_3_ group. The band at 1385 cm^−1^ is characteristic of the crystalline state and corresponds to the stretching of CH_3_ in PLLA and PHB. The intensity of the bands at 1385 and 1224 cm^−1^ for the crystalline segments of both PLLA and PHB were greater for the composites 5, 6 and 7 compared with pure PLLA; therefore, the addition of PHB and CNC made the crystallization of PLLA easier.

Some peaks appear in the PLLA at 735, 844 and 921 cm^−1^ and then disappear or weakly existing in biocomposites 5, 6 and 7. These peaks shift to lower frequency from 4.07 × 10^8^ to 3.98 × 10^8^ Hz, at 3.54 × 10^8^ to 3.44 × 10^8^ Hz, at 3.19 × 10^8^ to 3.13 × 10^8^ Hz and its intensity changes can be observed in these spectra. The curves at 753, 866 and 956 cm^−1^ appear in the pure PLLA and their composites and its intensity is greater in comparison with intensity of pure PLLA. Two bands appear at 870 and 753 cm^−1^. These two bands can be attributed to the amorphous and crystalline phases of PLLA, respectively. The band at 866 cm^−1^ shifts to the lower wavenumbers, and it becomes sharper by improving the crystallization process. The C–O stretching modes of the ester group appear at 1224 cm^−1^ and the C–O–C asymmetric mode appears at 1085 cm^−1^. In pure PLLA and their composites appears bands at 956 and 921 cm^−1^, these peaks are characteristic of the helical vibrations with the CH_3_ rocking modes. The band at 921 cm^−1^ is used to exam the kinetics of 10_3_ helix formation. With increasing the polymer crystallization, the peak heights at 1445, 1224, and 921 cm^−1^ are designed as a function for the crystallization. The carbonyl group C=O appeared broad, as well as the peaks at 735 to 753, 844 cm^−1^ moved to 866, 923 to 956 cm^−1^, respectively. These bands became broad in PLLA biocomposites as a result of a change in C=O that was directly attributed to intermolecular interactions, due to hydrogen bond interaction between the carbonyl groups of PLLA, PHB, CNC and TBC. The peak at 1640 cm^−1^ corresponds to the O-H bending of absorb water. It is well known that water is absorbed in the cellulose molecules and it is difficult to be completely removed due to the interaction of water with cellulose. This peek appears in both PLLA and CNC and as shoulder in biocomposites 5, 6 and 7. While another peaks at 1227 cm^−1^ for C-OH bending.

Wave number or wave number in the physical sciences is belonging to the wave. In spectroscopic uses wavenumber with the unit (cm^−1^). It is known that the unit of frequency is cycles per second or Hz. It is equal to the number of wave cycles in 3*10^10^ cm (the speed of light in one second), i.e. wave number = Hz/speed of light = (1/sec)/(cm/sec) = 1 /cm or cm^−1^. FT-IR 6 (C) verified that the carbonyl group C=O appeared broad and of some peaks in the PLLA composites shifted from 3.98 × 10^8^ to 4.07 × 10^8^ Hz, at 3.54 × 10^8^ to 3.44 × 10^8^ Hz, at 3.19 × 10^8^ to 3.13 × 10^8^ Hz.

#### Stress-strain behavior

**Figure 7:**
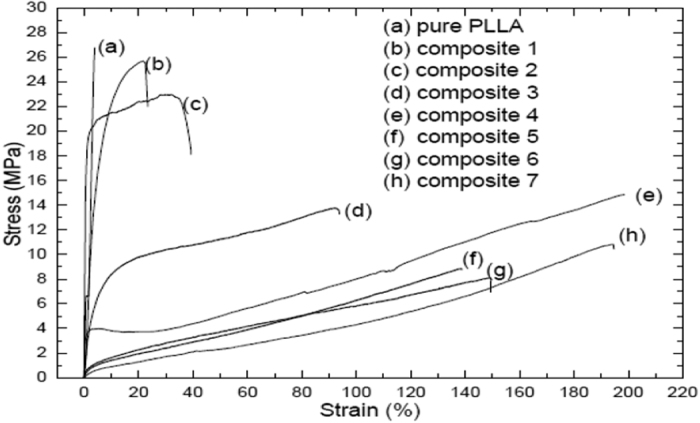
Stress-Strain curves of PLLA and its biocomposites at room temperature with speed 5 mm/min.

To improve the elongation at break of PLLA, the T_g_ of PLLA must be lower or near than the testing temperature and the size of the spherulites must be smaller (1–10 μm) together. If the T_g_ is lower than the testing temperature, the movement of the chains segments increases. The tensile tester of PLLA and its biocomposites was tested; the stress–strain curves are shown in Fig. 7. If we add PHB and CNC to PLLA matrix, the size of spherulite is small in comparison to pure PLLA, The addition PHB, CNC and TBC make PLLA like rubbery material. If we add small amount of PHB and plasticizer only to PLLA matrix, the material become very flexible and elongation at break stretches to 204%, therefore the addition of the plasticizer is important. By add the PHB, plasticizer and CNC or MCC together to PLLA matrix, the elongation at break decreases to 150%. The control in this research is pure PLLA without any additives for all samples. PLLA is brittle while PLLA biocomposites containing PHB, CNC or MCC and TBC together are ductile. A polymer is considered brittle, if its elongation at break is less than 15%. There is a clear difference when adding PHB, MCC and TBC to PLLA the stress becomes decreasing (25–14) MPa and strain increasing (22–80%) in comparison to pure PLLA. But when adding PHB, CNC and TBC to PLLA together becomes the stress decreasing (8–10 MPa) and strain increasing (140–190%) more in comparison to pure PLLA. When adding PHB and TBC only to PLLA, the stress (15 MPa) is decreased and strain (205%) is increased more in comparison to pure PLLA. The effect of micro- and nano-cellulose content on the mechanical properties of PLLA composites was studied. It was obtained and evaluated the tensile strength and elongation at break, for MCC and CNC. The results showed that the tensile strength decreased and elongation at break increased. It is found too that the content of CNC ratio is very smaller in comparison with the content of MCC in PLLA matrix.

**Figure 8:**
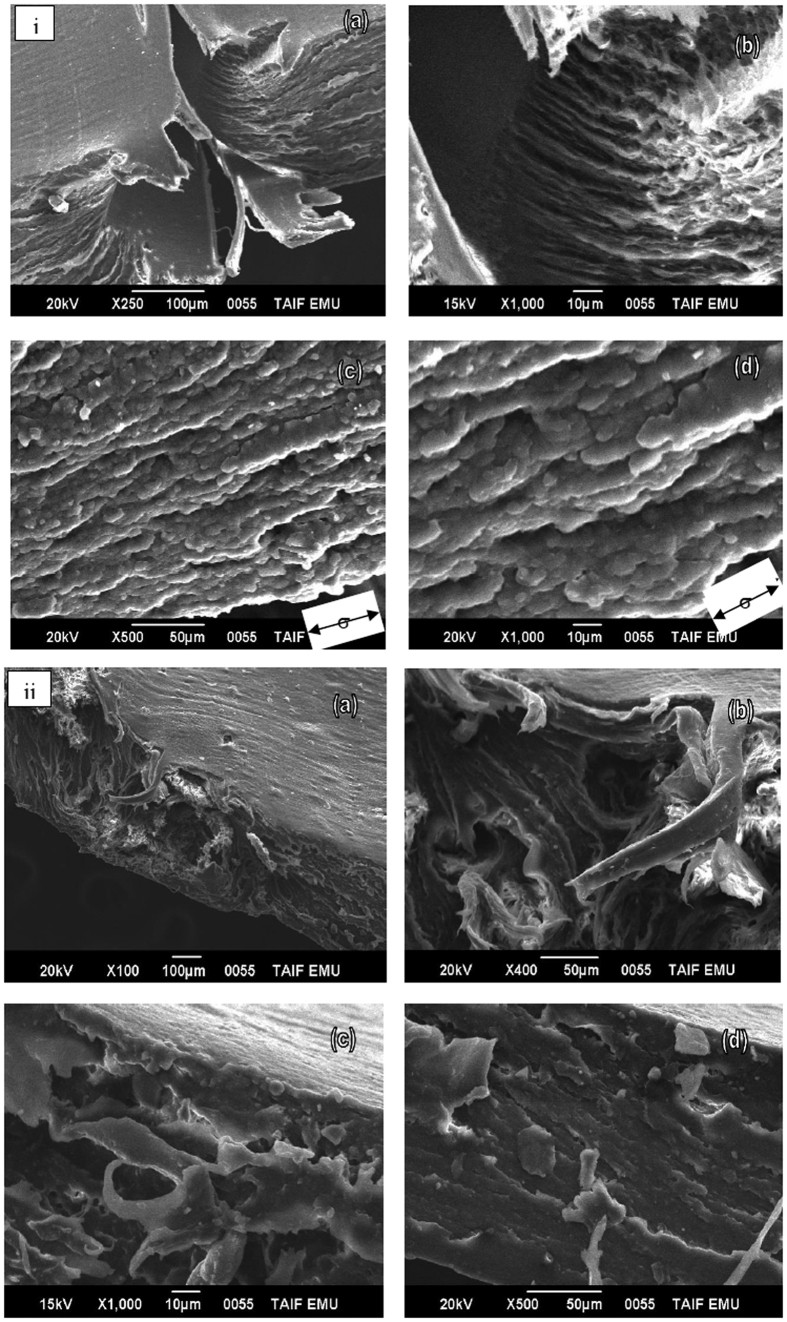
SEM micrographs fracture surface structure obtained from cold drawn film from biocomposites 4 (**i**) (a,b,c and d) and 7 (**ii**) (a,b,c and d).

Toughness is integration of the area under the stress-strain curve. toughness shows how much energy a material can absorb before breaking. Tough material must be both strong and ductile. For example, brittle materials (like pure PLLA) that are strong and the ductility are not tough. Blend 1 and 2 with low strengths are also not enough tough. Blend 3 to 7 with low strengths and enough tough. PLLA has a large strength of 27 MPa and an elongation at break of 6%. With the addition of PHB, CNC or MCC and TBC, the stiffness of PLLA is reduced while the elongation at break increases by 6–205%; the strength decreases to 27–8 MPa. A decrease in T_g_ and an decrease in spherulite size results in an increase of the elongation at break. Fig. 8 shows a SEM study of broken surfaces after cold drawn biocomposites 4 and 7; the composite samples elongate parallel to the stretching direction and produce fibril structures with elongation (see Fig. 8). The deformation takes place by shearing of crystals.

## Conclusions

Biocomposites from bioplastics are great interest for packaging materials due to its biodegradation and improved the elongation at break. The study of the thermal, crystallization behavior and tensile properties of PLLA biocomposites with PHB, CNC and plasticizer (TBC) were investigated by DSC, POM, TEM, SEM, WAXD and tensile testing. It can be observed that the T_g_, T_cc_ and T_m_ peaks shift towards lower temperatures with the addition of TBC, PHB and CNC together in PLLA matrix. POM demonstrated that pure PLLA had large spherulites, whereas all the biocomposites had small spherulites and the nucleation density of composites was enhanced compared with pure PLLA. DSC results indicated that the presence of PHB and CNC in PLLA matrix accelerated the crystallization process of biocomposites compared to PLLA. FT-IR revealed that the ester group C=O are broad and some peaks appear in the PLLA at 735, 844 and 921 cm^−1^ and then disappear in biocomposites 5, 6 and 7. These peaks shift from 3.98 × 10^8^ to 4.07 × 10^8^ Hz, at 3.54 × 10^8^ to 3.44 × 10^8^ Hz, at 3.19 × 10^8^ to 3.13 × 10^8^ Hz and its intensity changes. A new peak appeared at 2θ = 13.5° for PHB, at 2θ = 14.6° for PLLA, whereas the peak at 2θ = 16.4° is dramatically increased with addition PHB and CNC. The peak appeared at 2θ = 19° for PLLA, the peak at 22.4° appeared for PLLA and CNC. The dispersed PHB and CNC acted as bio-nuclei in PLLA matrix to help the crystallization rate and reduce the spherulite size, and therefore improve the the elongation at break from 6% for pure PLLA to more than 140–190% for the composites with CNC. PLLA biocomposites are suitable for packaged plastics especially in food sectors for deep drawing articles.

## Experimental Section

### Materials

Poly lactic acid (PLLA), Poly (R)-3-hydroxybutyrate (PHB), Tributyl citrate (TBC), a plasticizer, and microcrystalline cellulose fiber (MCC) were purchased from Sigma-Aldrich Chemical Ltd.

### Preparation of cellulose nanocomposites

MCC (1 g) was hydrolyzed in distilled water (300 mL) with magnetic stirring for 15 days at room temperature without use any acid (green method). Every 3 hours the suspension is heating for 30 minutes at 120 degree three time in the day. The suspension was then placed in an ice bath and stirred mechanically at 0 °C every 10 minutes. The CNC suspension was dried in a metal petri dish in an oven at 120 °C; the water evaporated and the CNC powder was collected.

### Preparation of compositions

The compositions of PLLA/PHB/TBC/CNC were prepared with different weight ratios as listed in Table 3. All composites were prepared by solvent casting of a film in a petri dish, and then dried at 60 °C for 24 hours to evaporate the solvent (chloroform). The biocomposites casting films were cut into small pieces and compression molded between two sheets of aluminum folder in a hydraulically heated press between 170 °C and 180 °C for 2 min without pressure and 1 minute with pressure 20 kN. After molding, the samples were cooled between two metal plates. At this temperature, thermal decomposition cannot occur. All samples are melted again at a temperature equal to T_m_ + 20 °C and at this temperature, thermal decomposition for DSC and POM does not happen.

### Measurements

#### Differential scanning calorimetry (DSC)

Thermal analysis was carried out with a differential scanning calorimeter (Shimadzu-DSC 50, Japan). Samples of 5 ± 0.1 mg were sealed in an aluminum sample pan under N_2_ and kept under a dry N_2_ atmosphere. DSC analysis was carried out from −50 °C to 190 °C with a heating and cooling rate of 10 °C min^−1^. Analysis of the second heating run was completed to determine the glass transition temperature (T_g_), melting temperature (T_m_) and cold crystallization temperature (T_cc_); the sample was held at 190 °C isothermally for 3 minutes to eliminate any thermal history, after which the sample was cooled to room temperature.

#### Polarized Optical Microscopy (POM)

The evolution of the microstructure of all composites was examined using a polarizing microscope (Nikon Eclipse E600, Japan) equipped with a hot-stage (Instec STC200, USA). A small amount of polymer was sandwiched between two microscope glass slides, placed on the heated stage, and melted at 200 °C, kept at this temperature for 3 minutes, and then cooled to the crystallization temperature (T_c_) of 80–120 °C. Samples were then maintained at this temperature as long as necessary for isothermal crystallization. POM micrographs were recorded using a digital camera.

#### Wide-angle X-ray diffraction (WAXD)

The crystalline phases were analyzed by wide-angle X-ray diffraction (PANalytical X’pert PRO diffractometer, Netherlands)) with Ni-filtered Cu-K_α_ radiation of λ = 1.54178 Å in the range of 5–35° at 40 kV. The WAXD data for the PLLA composites were obtained at room temperature (~25 °C) with a scan rate of (2°) 2θ min^−1^. Films were cut into rectangular pieces (4 cm^2^) and mounted on the sample holder for analysis.

#### Transmission electron microscopy (TEM)

The shape and size of CNC in a PLLA matrix was analyzed using a transmission electron microscopy (JOEL, model JEM1011) operating at 100 kV. Samples were prepared using chloroform as the solvent. One drop of the solution containing CNC and polymer was placed on a copper grid, the samples were prepared and then dried, placed into the TEM.

#### Fourier Transform Infrared Spectroscopy (FT-IR)

Infrared spectra in reflection mode were recorded with by Fourier transform infrared spectroscopy (FT-IR 6100 Jasco spectrometer, Japan) in the wavenumber range of 600–4000 cm^−1^. The films from all composites were cut into rectangular pieces (4 cm^2^) and spectra were recorded at room temperature.

#### Mechanical analysis

Uniaxial tensile tests were performed with dog bone specimens. All samples for testing in the tensile test device were cut in a dumb-bell shape (Dumb Bell Ltd SDL-100 Japan). Sample thickness was measured using a micrometer. Tensile tests were performed at room temperature, at a crosshead speed of 1 mm · min^−1^, using a Shimadzu universal testing machine equipped with a 10 kN load cell and interfaced with a computer. Five specimens of each formulation were tested and the average values are reported. From the correlation between stress σ (in Pa) and elongation ε (in %), *ε* = (L_0_ − L)/L, L_0_ = original length, L = length after elongation. At the end, the fracture surface of the electrospun samples was studied by SEM.

## Additional Information

**How to cite this article:** El-hadi, A. M. Increase the elongation at break of poly (lactic acid) composites for use in food packaging films. *Sci. Rep.* **7**, 46767; doi: 10.1038/srep46767 (2017).

**Publisher's note:** Springer Nature remains neutral with regard to jurisdictional claims in published maps and institutional affiliations.

## Figures and Tables

**Table 1:** Thermal characteristics of PLLA and its composites with different contents as determined by the second heating scan (Fig. 1).

Samples	T_g_ (°C)	T_cc_ (°C)	T_onset_ (°C)	T_m1_ (°C)	T_m2_ (°C)
Pure PLLA	60	106	82	156	168
Biocomposite 1	42	106	95	153	164
Biocomposite 2	38	103	88	149	163
Biocomposite 3	32	100	70	147	161
Biocomposite 4	27	91	81	145	160
Biocomposite 5	23	96	83	145	158
Biocomposite 6	18	94	75	141	157
Biocomposite 7	17	91	71	141	156

Glass transition temperature (T_g_), cold crystallization temperature (T_cc_) of composites, (T_m1_) first melting temperature, second melting temperature (T_m2_).

**Table 2:** Estimation of Crystallite Size of PLLA Composites.

Sample	Diffraction peak (hkl)	2θ (degree)	d (nm)	L_hkl_ (nm)
Composite 1	010	13.418	0.659	25.14
	110	16.893	0.534	16.15
	203	18.89	0.469	20.23
	015	22.31	0.398	16.56
Composite 2	010	13.45	0.658	26.39
	110	16.65	0.532	16.15
	203	19.04	0.466	13.90
	015	22.29	0.398	20.36
Composite 3	010	13.44	0.658	33.5
	110	16.65	0.532	20.23
	203	18.99	0.467	24.12
	015	22.29	0.398	20.39
Composite 4	010	13.45	0.657	29.89
	110	16.68	0.531	19.24
	203	19.07	0.465	12.63
	015	22.36	0.397	13.97
Composite 5	010	—	—	—
	110	16.55	0.535	23.7
	203	19.04	0.466	17.83
	015	22.25	0.399	19.53
Composite 6	010	—	—	—
	110	16.61	0.533	28.29
	203	19.01	0.466	21.39
	015	22.25	0.399	21.5
Composite 7	010	—	—	—
	110	16.64	0.532	30.5
	203	18.90	0.469	25.25
	015	22.25	0.399	21.5

**Table 3:** Percentage weight compositions of the different ratios from poly lactic acid (PLLA), polyhydroxybutyrate (PHB), tributyl citrate (TBC), micro crystalline cellulose (MCC) and cellulose nano crystal (CNC).

Composites	PLLA	PHB	TBC	MCC	CNC
Composite 1	65	10	15	10	—
Composite 2	57.5	10	20	12.5	—
Composite 3	50	10	25	15	—
Composite 4	70	10	20	—	—
Composite 5	74.90	10	15	—	0.10
Composite 6	72.35	10	17.5	—	0.15
Composite 7	69.80	10	20	—	0.20

## References

[b1] Song J. H., Murphy R. J., Narayan R. & Davies G. B. H. Biodegradable and compostable alternatives to conventional plastics. Phil. Trans. R. Soc. B 364, 2127–2139 (2009). 19528060 10.1098/rstb.2008.0289 PMC2873018

[b2] Auras R., Harte B. & Selke S. An overview of polylactides as packaging materials Macromol. Biosci. 4, 835–844 (2004). 15468294 10.1002/mabi.200400043

[b3] Robert J. L. & Aubrecht K. B. J. Ring-opening polymerization of lactide to form a biodegradable polymer. Chem. Educ. 85, 258–260 (2008).

[b4] Jain R. A. The manufacturing techniques of various drug loaded biodegradable poly(lactide-co-glycolide) (PLGA) devices. Biomaterials 21, 2475–2490 (2000). 11055295 10.1016/s0142-9612(00)00115-0

[b5] Ikada Y. & Tsuji H. Biodegradable polyesters for medical and ecological applications. Macromolecules Rapid Communication 21, 117–132 (2000).

[b6] Mallet B., Lamnawar K. & Maazouz A. Improvement of blown film extrusion of poly (Lactic Acid): structure–processing–properties relationships. J. Eng. Sci. 54, 840–811 (2014).

[b7] Yasuniwa M., Iura K. & Dan Y. Melting behavior of poly(L-lactic acid): Effects of crystallization temperature and time. Polymer 48, 5398–5407 (2007).

[b8] Kaito A., Iwakura Y. Li. Y. & Shimizu H. Effects of chain configuration on UCS behavior in blends of poly(L-lactic acid) with tactic poly(methyl methacrylate). J. Polym. Sci. Part B: Polym. Phys. 46, 1376–1389 (2009).

[b9] Pan P., Zhu B., Kai W., Dong T. & Inoue Y. Effect of crystallization temperature on crystal modifications and crystallization kinetics of poly (L‐lactide). J. Appl. Polym. Sci. 107, 54–62 (2008).

[b10] Kimura Y. Poly (p-dioxanone) and its copolymers polymer journal 41, 797–353 (2009).

[b11] Gentile P., Chiono V., Carmagnola I. & Hatton P. V. An overview of poly(lactic-*co*-glycolic) acid (PLGA)-based biomaterials for bone tissue engineering Int. J. Mol. Sci. 15, 3640–3659 (2014). 24590126 10.3390/ijms15033640 PMC3975359

[b12] Aslan S., Calandrelli L., Laurienzo P., Malinconico M. & Migliaresi C. Poly (D,L-lactic acid)/poly (∈-caprolactone) blend membranes: preparation and morphological characterisation J. Mater. Sci. 35, 1615–1622 (2000).

[b13] Bhatia A., Gupta R. K., Bhattachary S. N. & Choi H. J. Compatibility of biodegradable poly (lactic acid) (PLA) and poly (butylene succinate) (PBS) blends for packaging application. Korea Australia Rheology Journal 19, 125–131 (2007).

[b14] Yao M. . Modification of poly(lactic acid)/poly(propylene carbonate) blends through melt compounding with maleic anhydride. Express Polym. Lett. 5, 937–949 (2011).

[b15] El-Hadi A. M. Effect of processing conditions on the development of morphological features of banded or non banded spherulites of poly(3-hydroxybutyrate) (PHB) and polylactic acid (PLLA) blends Polym. Eng. Sci. 51, 2191–2202 (2011).

[b16] Tsuji H., Sawada M. & Bouapao L. Biodegradable polyesters as crystallization-accelerating agents of poly(l-lactide) ASC applied. Materials and interfaces 1, 1719–1730 (2009). 10.1021/am9002759 20355788

[b17] Hu Y., Sato H., Zhang J., Noda I. & Ozaki Y. Crystallization behavior of poly(l-lactic acid) affected by the addition of a small amount of poly(3-hydroxybutyrate). Polymer 49, 4204–4210 (2008).

[b18] Abdelwahab M. A. . Thermal, mechanical and morphological characterization of plasticized PLA–PHB blends Poly. Degrad. and Stab. 97, 1822–1828 (2012).

[b19] Yang X., Clénet J., Xu H., Odelius K. & Hakkarainen M. Two step extrusion process: from thermal recycling of PHB to plasticized PLA by reactive extrusion grafting of PHB degradation products onto PLA chains. Macromol. 48, 2509–2518 (2015).

[b20] Zhang M. & Thomas N. L. Blending polylactic acid with polyhydroxybutyrate: the effect on thermal, mechanical, and biodegradation properties Adv. Polym. Technol. 30, 67–75 (2011).

[b21] Armentano I. . Processing and characterization of plasticized PLA/PHB blends for biodegradable multiphase systems. Express Polym. Lett. 9, 583–596 (2015).

[b22] Zhang L., Xiong C. & Deng X. Miscibility, crystallization and morphology of poly(β-hydroxybutyrate)/poly(d,l-lactide) blends. Polymer 37, 235–241 (1996).

[b23] Mathew A. P., Oksman K. & Sain M. Mechanical properties of biodegradable composites from poly lactic acid (PLA) and microcrystalline cellulose (MCC) J. Appl. Polym. Sci. 97, 2014–2029 (2005).

[b24] Sundar S., Sain M. & Oksman K. J. Thermal characterization and electrical properties of Fe-modified cellulose long fibers and micro crystalline cellulose. Thermal Analysis Calorimetry 104, 841–847 (2011).

[b25] Dai X. . Fabricating highly reactive bio-based compatibilizers of poxidized ctric acid to improve the flexural properties of polylactide/microcrystalline cellulose blends. Ind. Eng. Chem. Res. 54, 3806–3812 (2015).

[b26] Haafiz M. K. M. . Properties of polylactic acid composites reinforced with oil palm biomass microcrystalline cellulose Carbohydr. Polym. 98, 139–144 (2013). 10.1016/j.carbpol.2013.05.069 23987327

[b27] Petersson L., Kvien I. & Oksman K. Structure and thermal properties of poly (lactic acid)/cellulose whiskers nanocomposite materials. Compos. Sci. Tech. 67, 2535–2544 (2007).

[b28] Iwatake A., Nogi M. & Yano H. Cellulose nanofiber-reinforced polylactic acid Compos. Sci. Tech. 68, 2103–2106 (2008).

[b29] Margaret J., Sobkowicz J. R., Keith W. D. & Herring G. A. M. J. Renewable cellulose derived carbon nanospheres as nucleating agents for polylactide and polypropylene. Ther. Analy. Calorm. 16, 131–140 (2008).

[b30] Dobrev T., Peren J. M., Perez E., Benavente R. & Garcia M. Crystallization behavior of poly(L-lactic acid)-based ecocomposites prepared with kenaf fiber and rice straw. Polym. Compos. 31, 974–984 (2010).

[b31] Sanchez-Garcia M. D. & Lagaron J. M. On the use of plant cellulose nano whiskers to enhance the barrier properties of polylactic acid. Cellulose 17, 987–1004 (2010).

[b32] Shi Q. . Mechanical properties and *in vitro* degradation of electrospun bio-nanocomposite mats from PLA and cellulose nanocrystals Carbohydr. Polym. 90, 301–308 (2012). 10.1016/j.carbpol.2012.05.042 24751045

[b33] Fortunati E. . Multifunctional bionanocomposite films of poly(lactic acid), cellulose nanocrystals and silver nanoparticles Carbohydr. Polym. 87, 1596–1605 (2012).

[b34] Frone A. N., Berlioz S., Chailan J. F. & Panaitescu D. M. Morphology and thermal properties of PLA–cellulose nanofibers composites Carbohydr. Polym. 91, 377–384 (2013). 23044146 10.1016/j.carbpol.2012.08.054

[b35] Navarro-Baena I., Kenny J. M. & Peponi L. Thermally-activated shape memory behaviour of bionanocomposites reinforced with cellulose nanocrystals. Cellulose 21, 4231–4236 (2014).

[b36] Qu P., Goa Y., Wu G. F. & Zhang L. P. Nanocomposite of poly (lactid acid) reinforced with cellulose nanofibrils. BioRes. 5, 1811–1823 (2010).

[b37] Kulinski Z., Piorkowska E., Gadzinowska K. & Stasiak M. Plasticization of Poly(l-lactide) with Poly(propylene glycol). Biomacrom. 7, 2128–2135 (2006). 10.1021/bm060089m 16827579

[b38] Cui L. . Rapid synthesis and characterization of chitosan-g-poly(D,L-lactide) copolymers with nonwovens of biodegradable PLA/acetyl tributyl citrate and copolyester blends. Appl. Polym. Sci. 125, E158–E167 (2012).

[b39] Burgos N., Martino V. P. & Jiménez A. Characterization and ageing study of poly(lactic acid) films plasticized with oligomeric lactic acid Poly. Degrad. Stab. 98, 651–658 (2013).

[b40] Courgneau C., Ducruet V., Avérous L., Grenet J. & Domenek S. Nonisothermal crystallization kinetics of poly(lactide)-effect of plasticizers and nucleating agent. Poly. Eng. Sci. 53, 1085–1098 (2013).

[b41] Martino V. P., Jiménez A. & Ruseckaite R. A. Processing and characterization of poly(lactic acid) films plasticized with commercial adipates. J. Appl. Polym. Sci. 112, 2010–2018 (2009).

[b42] Scatto M. . plasticized and nano filled poly(lactic acid)-based cast films: Effect of plasticizerand organoclay on processability and final properties. J. Appl. Polym. Sci. 127, 4947–4953 (2013).

[b43] Pilin I., Montrelay N. & Grohens Y. Thermo-mechanical characterization of plasticized PLA: is the miscibility the only significant factor? Polymer 47, 4676–4682 (2006).

[b44] Pluta M. Morphology and properties of polylactide modified by thermal treatment, filling with layered silicates and plasticization. Polymer 45, 8239–8250 (2004).

[b45] Xiao H., Yang L., Ren X., Jiang T. & Yeh J. T. Kinetics and crystal structure of poly(lactic acid) crystallized non isothermally: Effect of plasticizer and nucleating agent. Poly. Compos. 31, 2057–2069 (2010).

[b46] Baiardo M. . Thermal and mechanical properties of plasticized poly(L-lactic acid). J. Appl. Polym. Sci. 90, 1731–1738 (2003).

[b47] Ljungberg N. & Wesslen B. Preparation and properties of plasticized poly (lactic acid) films. Biomacrom. 6, 1789–1796 (2005). 10.1021/bm050098f 15877406

[b48] Jacobsen S. & Fritz H. G. Plasticizing polylactide – the effect of different plasticizers on the mechanical properties. Polym. Eng. Sci. 39, 1303–1310 (1999).

[b49] Martino V. P., Jiménez A. & Ruseckaite R. A. Processing and characterization of poly(lactic acid) films plasticized with commercial adipates J. Appl. Polym. Sci., 112, 2010–2018 (2009).

[b50] Lemmouchi Y. . Plasticization of poly(lactide) with blends of tributyl citrate and low molecular weight poly(D,L-lactide)-b-poly(ethylene glycol) copolymers. Eur. Poly. J. 45, 2839–2848 (2009).

[b51] El-Hadi A. M. Development of novel biopolymer blends based on poly(l-lactic acid), poly((R)-3-hydroxybutyrate), and plasticizer. J. Eng. Polym. Sci. 54, 1394–1402 (2014).

[b52] El-Hadi A. M. The effect of annealing treatments on spherulitic morphology and physical ageing on glass transition of poly lactic acid (PLLA). Materials Sciences and Applications 2, 439 (2011).

[b53] Zhang J., Elder T. J., Pu Y. & Ragauskas A. J. Facile synthesis of spherical cellulose nanoparticles. Carbohydrate Polymers 69, 607–611 (2007).

[b54] Lu P. & Hsieh Y. L. Preparation and properties of cellulose nanocrystals: rods, spheres, and network Carbohydr. Polym. 82, 329–336 (2010).

[b55] Wang N., Ding E. & Cheng R. Thermal degradation behaviors of spherical cellulose nanocrystals with sulfate groups. Polymer 48, 3486–3493 (2011).

[b56] Li X., Ding E. & Li G. A. method of preparing spherical nanocrystal cellulose with mixed crystalline forms of cellulose I and II Chinese. Journal of Polymer Science 19, 291–296 (2001).

[b57] Cheng M. . Efficient extraction of carboxylated spherical cellulose nanocrystals with narrow distribution through hydrolysis of lyocell fibers by using ammonium persulfate as an oxidant. J. Mater. Chem. A 2, 251–258 (2014).

[b58] Santis P. D. e. & Kovacs A. Molecular conformation of poly(S-lactic acid). Biopolymers 6, 299–306 (1968). 5641931 10.1002/bip.1968.360060305

[b59] Hoogsteen W., Postema A. R., Pennings J., ten Brinke G. & Zugenmaier P. Crystal structure, conformation and morphology of solution-spun poly(L-lactide) fibers. Macromol. 23, 634–642 (1990).

[b60] Furukawa T. . Structure, Dispersibility, and Crystallinity of Poly(hydroxybutyrate)/Poly(l-lactic acid) Blends Studied by FT-IR Microspectroscopy and Differential Scanning Calorimetry. Macromol. 38, 6445–6454 (2005).

